# The influence of voice familiarity and linguistic content on dogs’ ability to follow human voice direction

**DOI:** 10.1038/s41598-023-42584-2

**Published:** 2023-09-26

**Authors:** Livia Langner, Sabina Žakelj, Henrietta Bolló, József Topál, Anna Kis

**Affiliations:** 1 Research Centre for Natural Sciences, Budapest, Hungary; 2 University of Ljubljana, Ljubljana, Slovenia; 3 ELTE-HUNREN NAP Comparative Ethology Research Group, Budapest, Hungary

**Keywords:** Coevolution, Social behaviour, Neuroscience, Cognitive neuroscience, Attention, Intelligence

## Abstract

Domestic dogs are well-known for their abilities to utilize human referential cues for problem solving, including following the direction of human voice. This study investigated whether dogs can locate hidden food relying only on the direction of human voice and whether familiarity with the speaker (owner/stranger) and the relevance of auditory signal features (ostensive addressing indicating the intent for communication to the receiver; linguistic content) affect performance. N = 35 dogs and their owners participated in four conditions in a two-way object choice task. Dogs were presented with referential auditory cues representing different combinations of three contextual parameters: the (I) ‘familiarity with the human informant’ (owner vs. stranger), the (II) communicative function of attention getter (ostensive addressing vs. non-ostensive cueing) and the (III) ‘tone and content of the auditory cue’ (high-pitched/potentially relevant vs. low-pitched/potentially irrelevant). Dogs also participated in a ‘standard’ pointing condition where a visual cue was provided. Significant differences were observed between conditions regarding correct choices and response latencies, suggesting that dogs’ response to auditory signals are influenced by the combination of content and intonation of the message and the identity of the speaker. Dogs made correct choices the most frequently when context-relevant auditory information was provided by their owners and showed less success when auditory signals were coming from the experimenter. Correct choices in the ‘Pointing’ condition were similar to the experimenter auditory conditions, but less frequent compared to the owner condition with potentially relevant auditory information. This was paralleled by shorter response latencies in the owner condition compared to the experimenter conditions, although the two measures were not related. Subjects’ performance in response to the owner- and experimenter-given auditory cues were interrelated, but unrelated to responses to pointing gestures, suggesting that dogs’ ability to understand the referential nature of auditory cues and visual gestures partly arise from different socio-cognitive skills.

## Introduction

Domestic dogs (*Canis familiaris*) are effective communicators, and the factors associated with the comprehension and understanding of communicative cues by dogs has gained increasing attention in recent years [e.g.^1^]. There is evidence suggesting that dogs possess highly developed social skills to effectively communicate with humans in a unique way^2^. Possibly as a result of our convergent evolution during domestication, dogs developed human-like social competence, not only to communicate but also to cooperate with humans^3^. Therefore, the dog is increasingly used as a model species for investigating the evolution of human-analogous social behaviour and cognition^3–5^. Moreover, the Domestication Hypothesis suggests that the domestication process enabled dogs to develop interspecific social sensitivity and thereby to effectively coordinate their actions with humans in a collaborative manner^5,6^.

Dogs appear to be highly skilled in understanding several forms of human referential communication (functions to inform the receiver about context), including pointing and gazing^7,8^ and they are also sensitive to the indicators of the human’s communicative intention such as eye contact and/or high-pitched dog-directed intonation pattern^2,9^. Moreover, a cornerstone of human to dog communication is the fact that the intention of the human informant can acquire communicative value if his/her referential gestures are preceded or accompanied by ostensive signals indicating the speaker’s communicative intention (such as eye contact and intonational cues)^10^. This idea is further supported by the finding that dogs’ response to pointing gestures can be influenced by the human communicator’s tone of voice^11^.

It has been also reported that dogs (both adult subjects and puppies) can recognize the directional (referential) nature of human vocal communication; they can follow the direction of sound (human voice), and thereby rely solely on auditory information to solve an object choice task^12^. Rossano and colleagues^12^ conducted four studies to test dogs’ tendency to follow the direction of a human experimenter’s voice without any visual cues (i.e. the experimenter instructed the dog from behind a barrier). In their research, serving as a basis for the present study, dogs were observed in three different conditions in a two-way choice task that required subjects to locate the box containing food. In the ‘referential auditory signal’ condition the experimenter called the dog’s attention while orienting towards the baited container, in the ‘non-referential auditory signal’ condition the experimenter turned her back to the boxes and in the ‘no auditory signal’ condition she did not produce any vocalization from behind the barrier. The group level of data analysis indicated that dogs are successfully able to follow human vocalization in a directional manner and find hidden food without relying on olfactory or visual communicative cues. These findings challenge the assumption that such abilities are only utilized conjointly with visual modalities^5,13^. However, it remains an open question whether dogs’ ability to understand the referential meaning of visual and auditory signals can be conceptualized as a single domain general skill (cognitive ability influencing performance over situations and tasks, such as language comprehension) (c.f.^12^) or whether these are independent abilities, which would resonate with earlier findings indicating for e.g. that head shape and the selection of certain breeds for visual communication co-varies with pointing-following success^14^.

Nevertheless, it is also reasonable to assume that both ostensive and referential characters of human vocal signals could play a significant role in dogs’ responses to auditory cues. One important indicator of ostension is the specific ‘high-pitched dog-directed’ intonation pattern (c.f. doggerel—^15^). It has recently been reported that dogs can discriminate between adult-directed and dog-directed intonation pattern and show preference towards dog-directed speech (DDS), and linguistic content (actual substance of words in the message) that is comprehensible by a canine addressee^16^. Interestingly, DDS appears to be highly similar to motherese, the so-called infant directed speech (IDS) containing specific acoustic and linguistic features^17^. An fMRI study analysed the effect of lexical information and intonation on dogs’ brain responses (using verbal praises and neutral words) and found a functional link between auditory and reward regions in dogs, analogous to humans, indicating the importance of emotional intonation in a communicative context. This led to the conclusion that dogs tend to rely on both lexical meaning and intonation in human speech processing^18^. It seems however, that neither prosody nor linguistic content alone is solely responsible for dogs’ response to auditory cues, suggesting that content and prosody account for this preference in a conjoint manner^19^. Further evidence also suggests that dogs can rely on vocal into-nation to solve object choice tasks, utilizing positive versus negative intonation as a social referencing cue^20^. However, a more recent study has shown that the effect of intonation on dogs’ behaviour is strongly influenced by the presence of visual directional gestures such as pointing ^8^. Namely, vocal intonation can serve a social referencing function for dogs only when auditory cues and visual referential gestures (pointing) are presented concomitantly.

Another potentially important factor in communicating with human partners is social familiarity which is based on dogs’ ability to recognise familiar people following prior association and thus significantly influences many aspects of human–dog interactions. In line with this it has been shown that dogs tend to initiate contact with their owner more often than with unfamiliar or even relatively familiar humans, as well as pay more attention to that specific person^21^. Moreover, the relationship with the owner affects not only the amount of attention given by the dog but might also affect dogs’ ability to learn socially from human partners [^22^; but see^23^]. Nevertheless, dogs appear to show more success in solving tasks when the activity involves a familiar human, and their better task performance possibly boils down to cognitive abilities as well as the quality of the relationship^24^. Consequently, speech content and social relationship also affects dogs’ information processing abilities. Dogs’ relationship with the human speaker affects neural responses associated with reward and motivational processes, especially when it comes to the dog’s owner and praising speech^25^. Furthermore, dogs can also differentiate between speaker identity, word familiarity and emotional prosody^18,26^.

The current study aims to explore whether dogs can locate hidden food in a two-way choice task relying only on auditory information. The novelty of our study lies in the intent to understand whether dogs are able to make a choice based solely on the direction of auditory information, and whether such ability is affected by the identity of the speaker. The aim was to investigate whether the direction of human voice and whether the familiarity of the speaker (owner or stranger) as well as the relevance of auditory signal features (ostensive cues used by the speaker to convey her communicative intention, linguistic content) together can affect dogs’ task performance. We hypothesized that (1) dogs would successfully follow the direction of human auditory cues to find the hidden treats (thus replicating findings by Rossano et al.^12^). Moreover, we assumed that (2) dogs would be the most successful when the auditory cues are given by the owner (as compared to an unfamiliar experimenter). We also hypothesized that (3) dogs would demonstrate more success in the relevant auditory information condition (addressing in a high-pitched, ‘informing’ voice; relevant linguistic content of the message given that is interpretable by the dog in functional terms) than in the irrelevant auditory information condition (use of neutral tone; the linguistic content of the speech is irrelevant).

## Method

### Ethics statement

This research as well as its experimental protocol were approved for carrying out the below described behavioural experiment on family dogs with owner participation under Ref. No. PEI/001/1057-6/2015 (by the National Animal Experimentation Ethics Committee). Research was done in accordance with the Guidelines for the use of animals in research described by the Association for the Study Animal Behaviour (ASAB) and with the Declaration of Helsinki, also in accordance with the ARRIVE guidelines.

### Subjects and procedure

Companion dogs (*Canis familiaris*) older than 1 year of age (M ± SD = 5.97 ± 2.93 years) and their owners participated in the study (N = 37); 12 males and 25 females. Subjects were recruited via the Family Dog Project database of dogs’ owners. The ownership of dogs differed from one year of age to the maximum age of subjects (min. 1 year to max. 12 years). The exact age data of 3 dogs, as well as the dogs’ breed were unknown. Two dogs were excluded from the analysis due to lack of participation in the four conditions (opting out of more than two conditions throughout the test) thus the final sample consisted of 35 dogs. The experiment was carried out at the behavioural laboratory of the Institute of Cognitive Neuroscience and Psychology, between December 2019 and February 2020. All owners volunteered to participate in the study and provided informed consent prior to the start of the experiment.

**Figure 1:**
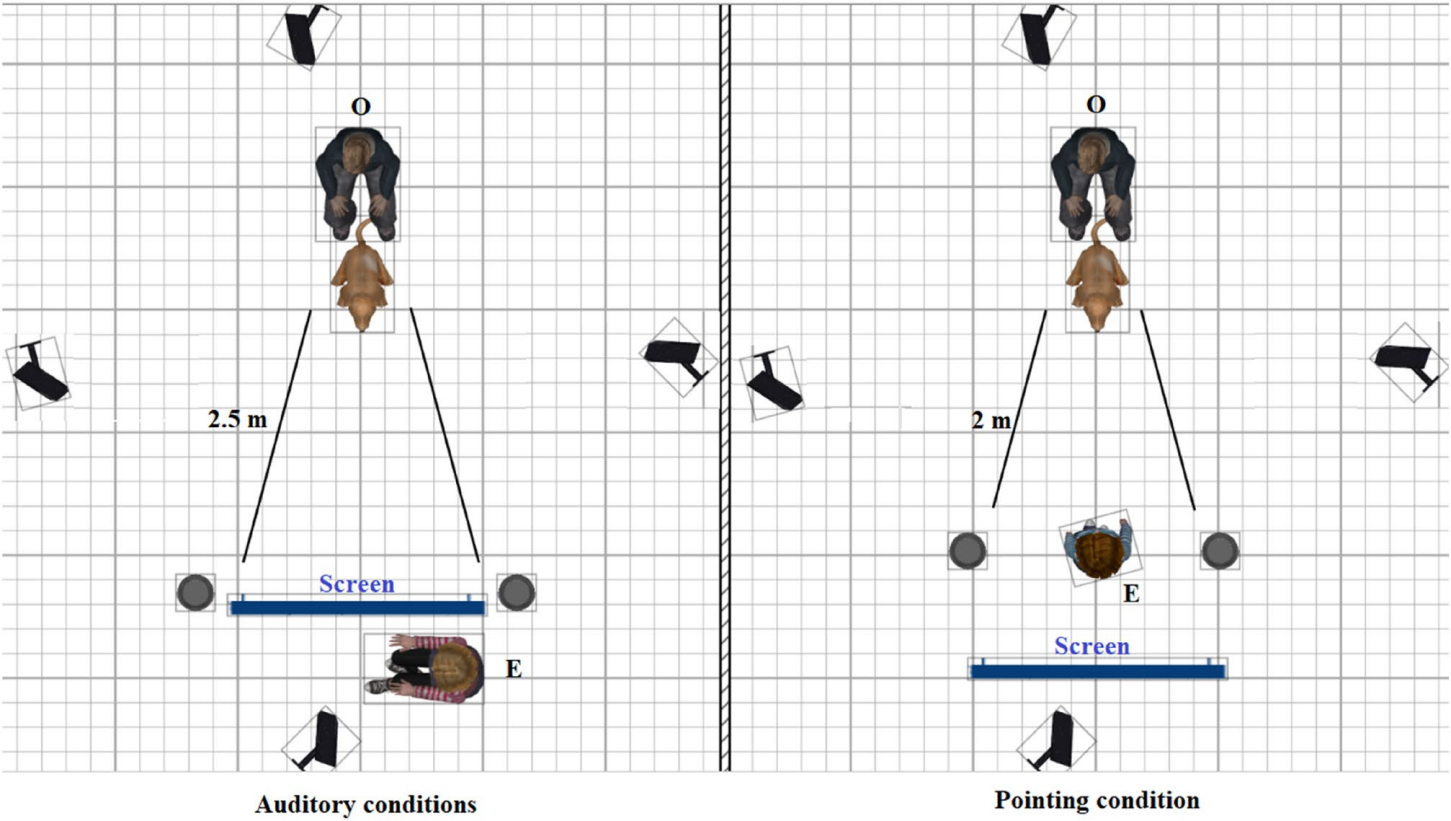
Schematic illustration of the experimental arrangement with the four camera angles. Caption (O) refers to the dog’s owner, while caption (E) refers to the experimenter.

Upon the entrance of the dog and the owner to the empty laboratory room, the dog was allowed to freely explore for a couple of minutes. Subjects were then familiarized with the experimental setup. First, one opaque container (15 cm deep, 15 cm in diameter) was placed one meter away from the dog with the treat (piece of dry dog food) on one occasion, and the experimenter waited until the dog approached it and ate the treat from the container. After this, the experimenter set the room according to the starting condition (the order of the conditions was randomised across subjects). Equipment provided throughout the tests included two containers, dog treats, a chair and a folding screen (1.2 m length × 1.5 m height)—see Fig. 1 for the experimental arrangement.

### Experimental conditions

Dogs participated in four experimental conditions, described as follows:

#### Potentially relevant auditory information/experimenter condition (PRAI/Exp)

For all three of the auditory conditions involved in the study, the baited container was indicated by the direction of the human’s voice. Both the experimenter and the owner were using the same Hungarian phrases during the demonstrations, but the experimenter had a foreign accent, which resulted in a slight pronunciation difference regarding the names of dogs and words used throughout the procedure. In an earlier study, the foreign experimenter’s pronunciation and accent did not interfere with the successful utilization of ostensive signals and the results remained replicable^27^. The ‘potentially relevant’ and ‘potentially irrelevant’ conditions further differed in the linguistic content of what was said, where the term potentially refers to the intended relevance/irrelevance of the content of the message (see Table 1. for a summary of procedural differences).

**Table 1:** Description of the four experimental conditions.

Condition	Demonstrator	Visibility of cuing	Attention getter	Visual referential cue	Signaling content	Auditory tone	Frequency of 1st/2nd/3rd/4th
Potentially Relevant Auditory Info./Experimenter (1st)	Experimenter (with foreign accent)	Behind folding screen (not fully visible)	[dog's name] + Look!	NA	It’s there! It’s yours! Look! It’s great!	High-pitched	12/9/4/12
Potentially Irrelevant Auditory Info./Experimenter (2nd)	Experimenter (with foreign accent)	Behind folding screen (not fully visible)	Knocking the two bowls together	NA	The weather is really warm today	Neutral	6/8/14/8
Potentially Relevant Auditory Info./Owner (3rd)	Owner	Behind folding screen (not fully visible)	[dog's name] + Look!	NA	It’s there! It’s yours! Look! It’s great!	High-pitched	9/7/9/12
Pointing/Experimenter (4th)	Experimenter (with foreign accent)	In front of folding screen (visible)	[dog's name] + Look!	Pointing	NA	NA	10/13/9/4

For this particular condition, the owner was sitting behind the dog in front of the folding screen, holding its collar. Some subjects remained on a leash, depending on the owners’ perceived ability to call back the dog. The experimenter hid a piece of dry kibble in one of the containers behind the folding screen out of sight to the dog. Still standing behind the folding screen, she turned towards the dog, looked at it and called out (“Look!”), saying its name. The experimenter waited until the dog was looking at her (above the folding screen), and then she crouched down behind the folding screen while holding one container in each hand, arms straight (the experimenter was not visible to the dog anymore by then). After putting the containers on the ground, she turned towards the baited one while crouched closer to the container with no treats (same as in Rossano et al.^12^). This was done in order to differentiate between a choice that follows the voice direction (this would lead to correctly choosing the baited location) versus a choice based on the source of the sound (this would lead to incorrectly choosing the non-baited location). The experimenter called out to the dog verbalizing relevant information using high-pitched dog-directed intonation pattern (“It’s there! It’s yours! Look! It’s great!”). When the call-out was finished, the dog was let free by the owner. The owner was allowed to use commands to facilitate the approaching of the container but not to indicate the direction to which the dog should go. Hiding of treats happened behind the folding screen out of sight to the dog.

#### Potentially irrelevant auditory information/experimenter condition (PIAI/Exp)

This condition was identical to the Potentially Relevant Auditory Information/Experimenter condition except that the experimenter did not use ostensive addressing (i.e. she only knocked the two bowls together to make a sound). Moreover, when she placed the two containers on the ground and crouched down closer to the empty one, she verbalized information irrelevant to the context (“The weather is really warm today.”) speaking in a monotone voice.

#### Potentially relevant auditory information/owner condition (PRAI/Own)

The procedure of the trials in this condition was identical to that of described above in Potentially Relevant Auditory Information/Experimenter condition, with the only exception, that the demonstration was performed by the owner. The experimenter sat down in front of the folding screen, holding the dog’s collar while it was sitting in front of her. The owner went behind the folding screen and closely followed the above-described procedure.

#### Pointing/experimenter condition (P/Exp)

This condition involved the baited container indicated by visual referential gesture throughout the trials. For this condition, the folding screen was put more backward contrasted to the auditory conditions, covering the area where the experimenter hid the treats. The owner sat down on the chair facing the folding screen with the dog sitting in the middle. The experimenter went behind the folding screen, out of sight to the dog and hid the treat in one of the bowls. After going back in front, she crouched down holding one bowl in each hand and put them on the ground while facing the dog. In standing position, she put both hands together and called out the dogs’ name for attention (“*dog’s name*, look!”). Utilizing the distal momentary pointing gesture (one hand, arms straight) she pointed towards the container (for 3 s) containing the treat while looking straight at the dog, taking her arm back to the starting position. The owner released the dog to approach one of the container but only after the experimenter’s arm was pulled back to the starting position.

A total number of 10 trials per condition were recorded with each subject, the position of the baited container (left or right hand side) was counterbalanced (5–5 trials), the individual order was assigned randomly. If the dog approached the baited container, it received the treat. However, if the selected container was the empty one, the experimenter removed the other (baited) container, and the dog was called back by the owner to prevent the dog from inspecting the other container. The experiment (4 × 10 trials in total) lasted about 20 min with a short break after the second condition. Two subjects refused to participate in the last condition, they were thus excluded from the analyses of these conditions. The order of the conditions was randomised at the group level (entire group of subjects) resulting in the frequencies listed in Table 1 for 1st/2nd/3rd/4th condition during testing (with one subject missing from the *PIAI*/*Exp* condition, thus resulting in a sum of 36 instead of 37 for frequencies of 3rd & 4th trials).

Experimental trials were recorded by four video cameras in fixed positions (see Fig. 1) and the behaviour of the subjects was analysed later. The aim was to investigate the effects of message content, speech intonation, and ostensive signals conjointly. The experimental arrangement was set up so that no distraction was present for the subjects in either the left or the right side of the room. The experimenter was a foreign national which might have influenced dogs’ performance in the *PRAI*/*Exp* and the *PIAI*/*Exp* conditions.

### Data analysis

Video recordings were coded by the first author (LL) frame-by-frame using Solomon Coder (beta 091110, ©2006 by András Péter). Coding was blind to expected results, e.g. tests were performed by a different person (SZ), and the person analysing the videos (LL) was only made aware of the study aims and hypotheses after coding was completed.

Based on the video recordings the following behavioural variables were extracted for each dog:

1. Latency to approach a container (s): defined as the time elapsed between the moment when the dog made the first step to approach the container and the moment when it touched one of the containers with its nose.
2. Choice (score): trials when the dog approached the correct (baited) container were scored as “1” and trials when the dog approached the incorrect (non-baited) container were scored as “0”.

Inter-rater reliability analysis was conducted to assess the coherence among independent observers of coding. For the left/right choice variable for 10% of the subjects (N = 4) all trials were re-coded by an independent coder (AK). Regarding correct choice, Cohen’s Kappa indicated a 100% agreement between coders (κ = 1). For the latency variable 1 randomly selected trial for N = 13 dogs (35% of the sample) was double coded so that one coder (AK) stopped the video either one frame before or one frame after the dog reached the pot (and noted down if the dog reached the pot—1, or haven’t reached the pot—0), then the second coder (LL) noted down for 1/0 for that frame assessing if the dog has reached the pot. With regards to latency, agreement between coders was also 100% (κ = 1).

If the dog did not approach any of the bowls within one-minute the trial was marked as ‘missed’. Missed trials were separated from the incorrect choices and were excluded from the analyses as missing value. Missed values appeared throughout the four conditions as follows: *P*/*Exp*: 7; *PIAI*/*Exp*: 11, *PRAI*/*Exp*: 3; *PRAI*/*Own*: 4. In the Spearman’s correlational analysis, missed values were indicated as incorrect trials, marked as zero. Chi square test indicated a non-significant tendency between the conditions including versus not including missed cases (*χ*^2^ (3) = 7.460; *p* = 0.059).

Statistical analysis involved a Generalized Linear Mixed Model (GLMM) to build the two separate statistical models for Choice and Latency of response as output variables with subject ID as a random factor. The aim of the analysis was to identify possible interactions among independent variables on two different levels (two-way interactions). The binomial generalized linear mixed model for Choice, included the following factors: condition (*P*/*Exp*; *PIAI*/*Exp*, *PRAI*/*Exp*; *PRAI*/*Own*), trial number (1–10), side of baiting (left/right), latency of response and their two-way interactions.

The generalized linear mixed model for Latency of response included the same four fixed factors (condition, trial number, side of baiting and choice) and their two-way interactions. Non-significant effects were systematically removed (stepwise, backward elimination) from both models. Statistical tests were two-tailed, the α value was set at 0.05.

Group level performance (total percent of correct choices) was contrasted to the 50% chance level, separately for the four conditions (one sample t-tests), in order to see which referential cues were followed successfully by the subjects. In parallel, individual dogs with above/at/below chance performance were also identified (binomial test, test proportion: 0.5). Spearman's correlations were conducted to analyse the relationship between performance in the four conditions. Shapiro–Wilk test indicated a non-normal distribution of data in the *P/Exp* condition (*p* > 0.05).

All statistical analyses were carried out using SPSS software package (v. 25.0).

## Results

Looking at the individual results (Fig. 4), binomial tests revealed that out of the N = 35 dogs N = 3 showed an above chance performance in the *PRAI/Exp* condition (N = 2 at 90% performance, *p* = 0.021 and N = 1 at 100%, *p* = 0.001 performance), while N = 33 dogs performed at chance, and N = 1 below chance (at 0%, *p* = 0.001). A non-parametric Friedman test was conducted to examine missed trials but missed trials did not differ significantly among the four conditions (*p* > 0.05). In the *PRAI*/*Own* condition N = 3 dogs showed an above chance performance (N = 3 at 90% performance, *p* = 0.021). All but one remaining individual’s performance were at chance level (< 80% & > 20%, *p* > 0.05) while N = 1 performed below chance (at 10%, *p* = 0.021). In the *PIAI/Exp* condition, N = 1 dog showed a below chance level performance (at 10%, *p* = 0.021), the remaining dogs performed at chance. All subjects performed at chance level in the *P*/*Exp* condition.

Contrasting the condition-by-condition group level results to the 50% chance level, Wilcoxon independent sample test indicated that correct choices in the *PRAI/Own* condition (Z(35) = − 3.219; *p* = 0.001), as well as the *PRAI*/*Exp* (Z(35) = − 2.037; *p* = 0.042), were significantly above chance level. Performance in the remaining conditions *(PIAI/Exp* and *P/Exp* conditions) did not differ from chance level (all *p* > 0.05).

**Figure 2:**
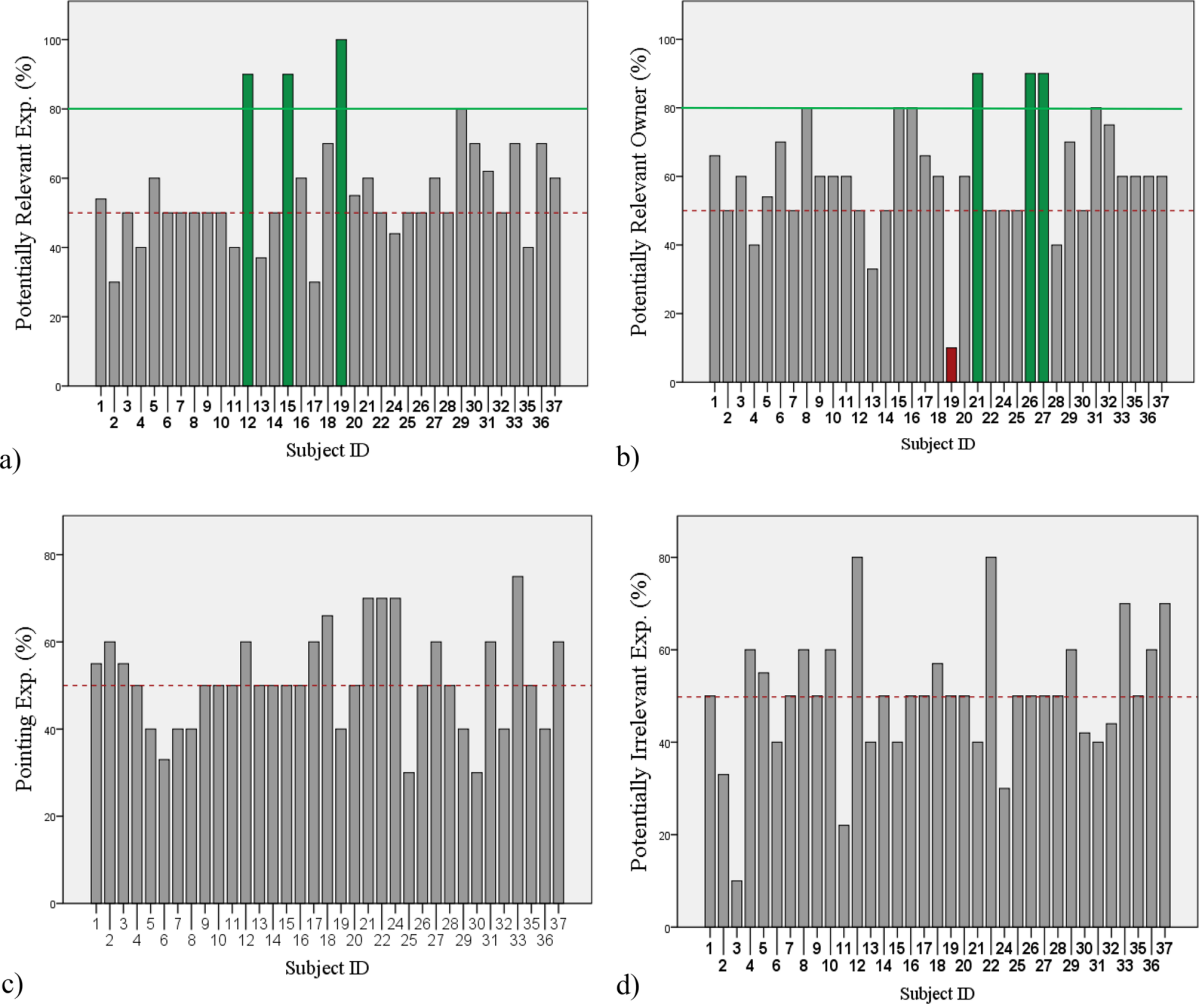
Percent of correct responses for each individual in the PRAI/Exp (**a**), PRAI/Own (**b**), P/Exp (**c**) and PIAI/Exp (**d**) conditions. Dashed line indicates 50% chance level. Individuals with an above chance performance are highlighted with green. Individuals with a below chance performance are highlighted with red.

Dogs' choice behaviour was significantly influenced by the main effect of Condition (binomial GLMM, F_3,75_ = 6.391; *p* = 0.001; Fig. 2). Most correct choices were made in the *PRAI/Own* condition (M = 60.4%; SD = 16.85), followed by the *PRAI*/*Exp* condition (M = 56.34%; SD = 16.07). Correct scores in the *P*/*Exp* condition were less frequent (M = 51.26%; SD = 11.45), and as expected, dogs scored the lowest in the *PIAI*/*Exp* condition (M = 49.80%; SD = 14.18). Post hoc pairwise comparison revealed a significant difference between the *PRAI/Own* and the *PIAI*/*Exp* condition (t_75_ = − 3.947; *p* < 0.0001). A significant difference was also revealed between the *PRAI*/*Own* and the *P*/*Exp* condition (t_75_ = − 3.603; *p* = 0.001). Moreover, a significant difference was revealed between *PRAI*/*Own* & the *PRAI*/*Exp* condition (t_75_ = − 1.993; *p* = 0.05).

Upon inserting missed values as incorrect choices into the model, as well as N = 2 dogs with partial results, the final results showed the same pattern as that of the original model (*PRAI*/*Own* condition (M = 59.24%; SD = 16.6); *PRAI*/*Exp* condition (M = 55.05%; SD = 18.64); *P*/*Exp* condition (M = 50.22%; SD = 14.34); *PIAI*/*Exp* condition (M = 47.95%; SD = 16.52).

We also found a significant main effect of Side (F_1,75_ = 5.003; *p* = 0.028) with dogs making more correct choices on their left side irrespective of test condition. Moreover, a significant interaction between Trial and Side was revealed (F_1,75_ = 5.027; *p* = 0.028) indicating that dogs made even more correct choices on the left side as the number of trials advanced. The main effect of Trial and Latency, as well as the rest of the interactions were non-significant (all *p* > 0.05).

**Figure 3:**
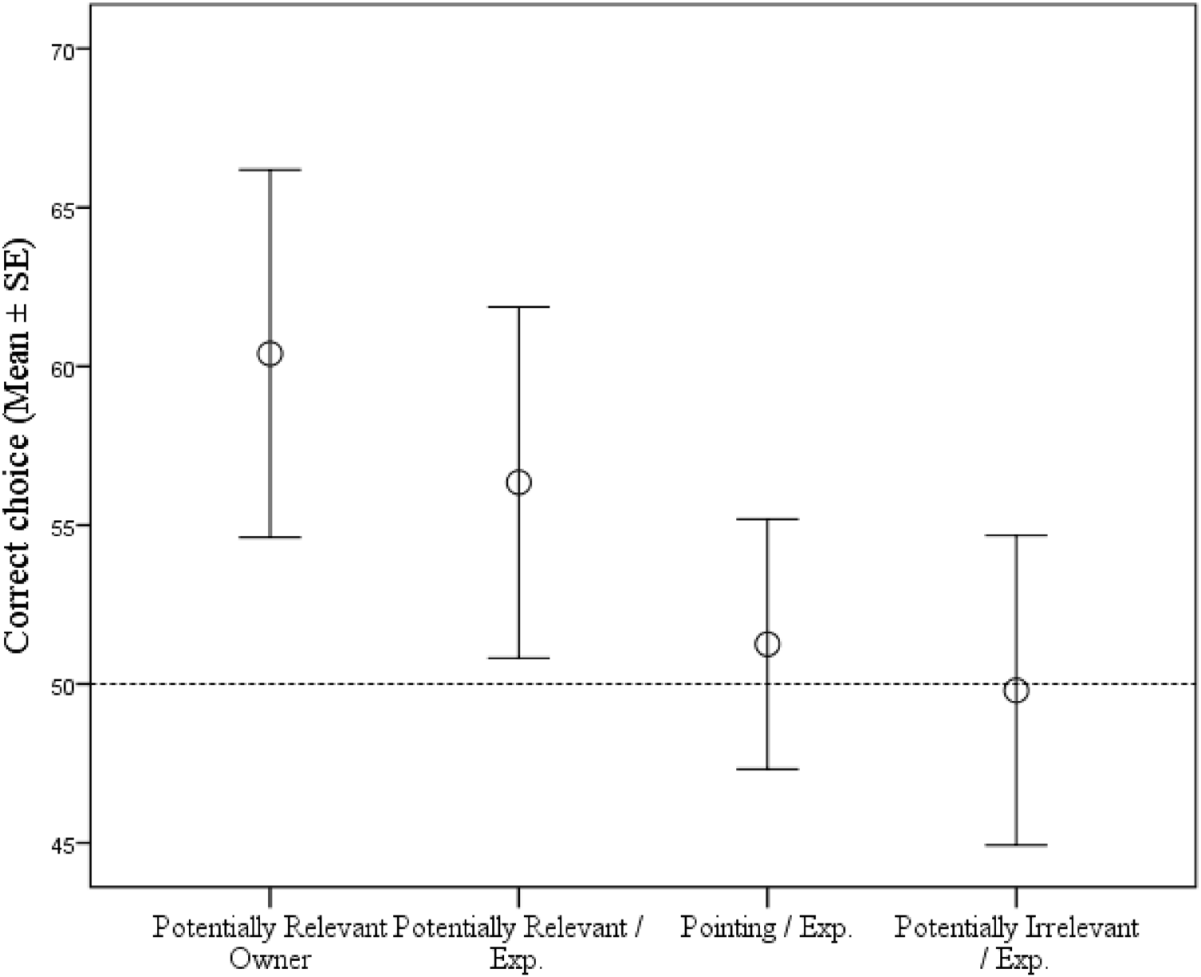
Mean ± SE percentage of subjects’ correct choices in the four conditions (out of 10 trials). Solid horizontal line denotes the 50% chance level.

**Figure 4:**
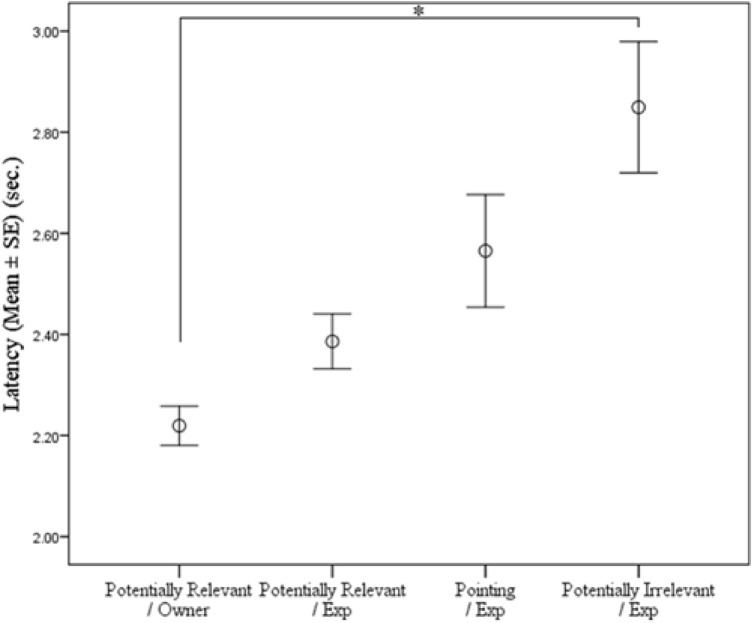
Subjects’ response latencies (mean ± SE) in the four conditions. * indicates significant difference at *p* < 0.05 level.

A significant main effect of Condition was also observed on Latency of response (F_3,1360_ = 8.722; *p* < 0.0001) with faster response times for the conditions with more correct choices (*PRAI*/*Own* < *PRAI*/*Exp* < *P*/*Exp* < *PIAI*/*Exp*). Post hoc pairwise comparison revealed a significant difference between the *PRAI*/*Own* and the *PIAI*/*Exp* condition (t_1360_ = − 4.884; *p* < 0.0001). A significant difference was also revealed between the *PRAI/Own* and the *P*/*Exp* condition (t_1360_ = − 2.681; *p* = 0.007), as well as between the *PIAI*/*Exp* and the *P*/*Exp* condition (t_1360_ = 2.200; *p* = 0.028). Moreover, a significant difference was also observed between the *Potentially Relevant-* and *PIAI/Exp* conditions (t_1360_ = − 3.595; *p* < 0.0001) (Fig. 3). The main effect of Correct choice, Trial, Side, and their interactions were non-significant (all *p* > 0.05) (Fig. 4).

Spearman’s correlation was conducted on the dataset where missed trials were included as values (incorrect responses). Analysing the possible relationship between individual responses to the different cues revealed a weak, but significant positive correlation between dogs’ performance in the *PIAI*/*Exp* and the *PRAI*/*Exp* conditions (r = 0.336, *P* = 0.028). No significant correlations were identified between the *PRAI*/*Own* condition and the *PRAI*/*Exp* condition (r = 0.201, *p* = 0.233) or the *PIAI*/*Exp* condition (r = − 0.062, *p* = 0.718), nor between the *P*/*Exp* condition and any of the auditory conditions (all *p* > 0.05). The above results are summarized in Table 2.

**Table 2:** Results of the four experimental conditions.

Condition	*PRAI*/*Own*	*PRAI*/*Exp*	*PIAI*/*Exp*	*P*/*Exp*
Correct choice (number) (Mean ± SD)	60.4 ± 16.85	56.3 ± 16.07	49.8 ± 14.18	51.3 ± 11.46
Latency (sec) (Mean ± SD)	2.3 ± 0.76	2.4 ± 1.01	3.0 ± 2.71	2.6 ± 2.03
Above chance level	N = 3	N = 3	N = 0	N = 0
Below chance level	N = 1	N = 0	N = 0	N = 0

## Discussion

Earlier research indicated that dogs are not only sensitive to cues signalling human communicative intent but also have some understanding of the referential character of directional gestures, such as pointing or gazing^2^. Although there is general agreement that these skills manifest themselves most prominently when a gesture performing a referential function is a visual sign^9^, there is also evidence that dogs comprehend the referential character of auditory signals (i.e. directional characteristics of human voice—^12^). Our findings confirm those of Rossano et al.^12^, who reported that dogs possess the capacity to solve object choice tasks by relying solely on the direction of auditory cues. But our study also revealed that dogs performed more successfully in conditions involving potentially relevant auditory cues (meaningful content with dog-directed intonation) by a socially relevant human companion (the owner). An earlier study by Kiss et al. concluded that dogs are more prone to interact in a fetching task upon receiving cues from a person similar to their owners^28^. Moreover, the average latency of response was also found to be the lowest in the owner-involved task. This finding is in line with those of earlier research concluding that dogs perform better when interacting with their owner than an unfamiliar experimenter e.g. in emotion recognition^29^, visual attention^22^, social referencing^30^ and dog–human interaction^21^ tasks. Others, however, have found no performance difference in a food choice task depending on the demonstrator being the owner versus the experimenter^31^. Dogs have also been shown^28^ to prefer a human partner similar to their owner in an unsolvable problem task—they were less prone to cooperate with an experimenter with strange motion pattern and language usage. In our study the experimenter had a foreign accent (non-native Hungarian which might have influenced dogs’ understanding of commands), thus this “out-group” effect might have contributed to the observed differences between conditions. Nevertheless, in our study, in the owner-involved condition, the experimenter was the one holding/releasing the dog throughout the trials, and dogs made the most correct choices in this condition. However, since in the owner condition dogs’ owners call out to the dog with the auditory cue, which could serve as a command for the dogs to reach their owners in a rapid manner, regardless of the specific cue provided. Consequently, as Schmidjell et al. found, owners’ have hardly any influence in deliberately directing dogs’ choice behaviour^32^.

Contrasting dogs’ performance in the voice-following condition to that of a Pointing condition (carried out by the experimenter) it was found that subjects performed somewhat worse in the latter, and surprisingly they did not differ from the chance level neither as a group, nor as individuals. This contrasts prior findings, which have been vastly replicated, and show that dogs are capable of following human distal momentary pointing^7,13^. Nevertheless, this result is not unprecedented, as others reported findings that a considerable proportion of individual dogs performed at chance level (N = 13 out of N = 24;^33^). Udell et al.^34^ also reported similar results, where dogs were presented with distal momentary pointing gestures and showed chance performance on the group level (although the latter results might have been at least partly due to the outdoor testing environment). Furthermore, in our study the experimenter presented the task with an accent unfamiliar to the dogs. An fMRI study confirmed^26^ that dogs are able to distinguish between familiar and unfamiliar languages as reflected in the activation of brain regions in the secondary auditory cortex. This together with the finding that dogs prefer to work with “in-group” humans (c.f.^28^) might explain their relatively poor pointing-following performance in our study. Importantly, in the present study all subjects participated in four different conditions, thus they completed a relatively large number of experimental trials (e.g. compared to previous pointing-following studies). This may have contributed to a lower level of success due to fatigue or motivation loss. Although no significant trial-effect was found in our dataset, the significant interaction between trial number and side chosen might indirectly indicate a change in dogs’ strategy as the test progressed (e.g. increased tendency for side preference).

Our main finding was that the tone of voice and the intonation of the message together emerged as a potential predictor of task performance, supported by the fact that dogs responded more rapidly to potentially context-relevant information in contrast to irrelevant auditory cues. These findings support earlier research stating that dogs following visual cues might be context-dependent and can be influenced by auditory signals and their intonation^11^. Nevertheless, it can be proposed that in our study dogs might have been able to estimate the direction of the head behind the folding screen and therefore the source-direction of information throughout the auditory conditions. It might be worth investigating further whether an additional condition involving visible head turning and relevant auditory information could result in similar (or even better) performance compared to the relevant experimenter and owner conditions.

Additionally, the intonation of the message conveying context-relevant information might also affected dogs’ performance, namely that the relevant auditory content and positive intonation have influenced dogs’ success. Investigating the effect of lexical and intonational differences on dogs’ processing of human speech separately, Andics et al.^18^ found that lexical representations can be separated from acoustic representations in dogs. Moreover, dogs are capable of distinguishing between natural speech and quilted speech (artificially scrambled speech where auditory regularities specific to speech are missing but spectral voice cues are intact) which might indicate a sensitivity to perceived naturalness of speech^26^. Therefore, further research should consider investigating the effect of context-relevant auditory information utilizing more varied intonational gestures regarding tone and intensity.

The effect of side also emerged as a significant predictor of dogs’ performance. An earlier study investigated the effect of emotional valence on dogs’ lateralized brain response, demonstrating the prevalent use of the right hemisphere in response to vocalization with negative emotional valence, and the use of the left hemisphere in response to positive vocal cues^35^. Furthermore, research has also shown that upon presentation of human spoken commands (higher salience of meaningful phonemic components), dogs turned their heads more to the right side (i.e. the left hemisphere is favoured during speech processing). However, a significant left-side bias (indicating right-hemispheric dominance in processing) was observed with increased suprasegmental vocal cues (higher salience of intonational components)^36,37^. This might also explain the effect of side in our results. Moreover, negative emotional valence can also facilitate avoidance behaviour, while positive emotions can exacerbate approaching in dogs^38^. Our results are partly in line with these findings, where approaching behaviour was expected in response to positive vocalization. However, the direction of the approaching behaviour is rather the opposite as in previous studies.

The results of individual performance suggest that comprehension of voice direction was apparent on the group level. Consequently, two dogs performed below chance level in the P*RAI*/*Exp* and *PIAI*/*Exp* conditions, which might indicate avoidance behaviour in response to auditory signals. It was found earlier^9^ that dogs avoid approaching a target object when they supposedly assume the experimenter’s interest in the object from ostensive gazing, and inference competitive interests. In the current study there were no below chance results in the case of the *P*/*Exp* condition, only in the voice following conditions. Interestingly, there was one subject whose performance was below chance level in the *PRAI*/*Own* condition, while above chance level in the *PRAI*/*Exp* condition. These results might indicate that this dog showed more profound separation anxiety when the owner was hidden behind the screen, and the dog’s increased anxiety might account for its poor performance.

Furthermore, dogs’ performance in response to experimenter-given auditory cues (relevant and irrelevant) were related to each other, but not to response to the experimenter’s pointing. This together might indicate that responsiveness to the direction of auditory cues stands as a trait-like ability of dogs, whereas responding to directional visual gestures differs from the ability of responding to the direction of auditory cues. This contradicts the implicit assumption^12^ that responding to referential cues might be a sole cognitive capacity and is instead in line with findings^14^ which have shown that the capacity to follow human pointing in dogs is related to selection for visual skills and head shape. Moreover, missed cases occurred the most frequently in the *PIAI*/*Exp*, which might suggest that understanding of the potentially irrelevant cue by dogs was the worst in this condition.

Consequently, an alternative hypothesis to our results could suggest that dogs in our study might relied on directional hearing in response to auditory cues, namely, that the echo from the sides of the experimental setting might influenced their choice of side. Gergely et al.^39^ found that dogs are able to differentiate between qualitatively different sounds and generalize directional cues in novel settings. Consequently, the above hypothesis could not explain the differences in the results of the *Potentially Irrelevant-* versus the *Potentially Relevant* auditory condition.

A limitation of the study lies in the fact that further conditions, namely a *PRAI*/*Own* condition would be interesting to test. The reason for emitting such condition was the possible tiring out/drop-out of dogs when participating in too many conditions. Nevertheless, the two conditions with the experimenter and only one with the owner allowed for the experimenter’s behaviour to be standardized to a high extent, while two conditions involving the owner could have resulted in procedural mistakes. Future research should consider testing a potentially irrelevant auditory condition involving the owner/ familiar person. A couple of studies have already provided strong evidence that in a pointing following task dogs cannot be influenced by their owners (who kept them before making a choice); e.g. pushing them toward a certain location is insufficient to affect their choice, even if the owners are instructed to actively try and influence the dogs’ response to human pointing^32,40^. One would assume that this result holds true for the voice following task as well, however it has not been directly tested yet. The question of whether holding the dogs’ collar would have an involuntarily effect on directing the dog remains to be studied. Furthermore, as referenced earlier, head shape and the selection of certain breeds might have a possible influence on dogs’ cognitive abilities to understand referential cues^14^. Future studies might consider taking head shape into consideration upon interpreting results testing cognitive abilities, especially in the case of mixed breed subjects.

In sum, the findings of our study indicate that task performance is not exclusively interconnected with or related to visual communicative cues such as the pointing gesture, and dogs are successful in following solely auditory signals for problem solving. Intonational and lexical cues, as well as the source of information appears to be influential with regards to performance (effect of owner versus unfamiliar experimenter), since dogs made more correct choices more frequently in conditions involving auditory information with relevant content as well as a familiar human. Our findings conclude on an important aspect regarding the potentials of human–dog communication and sociocognitive mechanisms of dogs. Human–dog communication has possibly evolved as a result of domestication, and dogs’ comprehension of auditory communicative signals can further underline the effect of this process. Future research should consider investigating the effect of context-relevant auditory information utilizing more varied intonational gestures, as well as the effect of auditory signals on wolves.

## Conclusions

The current study investigated the extent dogs are able to utilize human communicative gestures, namely auditory and visual signals combined with referential cues. Our results confirmed that dogs are able to solve object choice tasks by relying on auditory cues. Moreover, findings revealed that the content of the message as well as the identity of the speaker (owner vs. unfamiliar experimenter) also affect dogs’ success, namely that performance was more successful in conditions involving potentially relevant auditory cues and a socially relevant speaker (owner). Furthermore, the tone of voice as well as the intonation of the message together emerged as a predictor of performance.

Our findings indicate that dogs’ performance is not exclusively related to visual signals, and that dogs are able to utilize auditory communicative cues for problem solving. Moreover, the source of information, as well as intonational and lexical cues influence dogs’ performance, highlighting on the special mechanisms of human–dog communication.

## Data Availability

The data presented in this study are available on request from the corresponding author.
